# Inclusion of key populations in clinical trials of new antituberculosis treatments: Current barriers and recommendations for pregnant and lactating women, children, and HIV-infected persons

**DOI:** 10.1371/journal.pmed.1002882

**Published:** 2019-08-15

**Authors:** Amita Gupta, Michael D. Hughes, Anthony J. Garcia-Prats, Katherine McIntire, Anneke C. Hesseling

**Affiliations:** 1 Department of Medicine, Division of Infectious Diseases, Johns Hopkins University School of Medicine, Baltimore, Maryland, United States of America; 2 Center for Biostatistics in AIDS Research, Harvard T. H. Chan School of Public Health, Boston, Massachusetts, United States of America; 3 Desmond Tutu TB Centre, Department of Paediatrics and Child Health, Faculty of Medicine and Health Sciences, Stellenbosch University, Tygerberg, South Africa

## Abstract

Amita Gupta and colleagues discuss priorities in clinical research aimed at improving tuberculosis prevention and treatment in pregnant women, children, and people with HIV.

**Summary points**

- Pregnant women, children < 15 years old and, HIV-infected persons contribute approximately 20% of the global tuberculosis (TB) burden, with an estimated 216,000, 1,000,000, and 1,040,000 cases each year, respectively, yet these populations are currently largely excluded from TB clinical trials, leading to suboptimal treatment and poor access to new therapeutics.
- Special considerations in these populations include specific TB disease spectrum and severity, lower sensitivity of commonly used TB diagnostic tests, potential differential drug dosing and treatment responses, drug–drug interactions, and challenges in acquiring high-quality data through clinical trials.
- To counter the automatic exclusion of pregnant and lactating women that currently pervades the TB trial landscape, early discussions among trialists, pharmaceutical companies, maternal–child clinical experts, ethicists, and regulatory bodies are needed to address risks, benefits, and compelling rationale for inclusion. Reconsenting women when pregnancy occurs on a trial to allow continuation of study drug by informed choice is a practical and valuable approach to expand the currently limited evidence base.
- Children tend to have less severe, often paucibacillary TB disease and may respond better to treatment than adults. Consequently, trials of shorter, less intense TB treatment regimens in children are needed; pharmacokinetic and safety studies should be initiated earlier and involve age groups in parallel rather than in an age-de-escalation approach. More rapid development of child-friendly drug formulations is needed.
- All HIV-infected populations, including those with advanced disease, who are likely to be the intended population of the TB therapy, should be involved in Phase IIb and/or Phase III trials, as appropriate, to maximize knowledge of treatment, toxicities, drug–drug interactions, and outcomes.

## Introduction

Globally, 10 million cases of active tuberculosis (TB) disease and 1.6 million TB-related deaths occurred in 2017 [1]. Pregnant and postpartum women, children < 15 years old, and HIV-infected persons account for 20% of the global TB burden, with an estimated 216,000, 1,000,000, and 1,040,000 cases each year, respectively [1,2]. Special considerations in these populations include TB disease spectrum and severity, lower diagnostic sensitivity, possible differential treatment responses, drug dosing and interactions, and challenges in acquiring high-quality data through clinical trials [3–5]. Without clear consideration of actual risks and benefits of trial participation, pregnant women have been uniformly excluded from TB therapeutic trials, especially for multidrug-resistant (MDR) TB [6,7], based on fears of harming the fetus and legal liability [8]. Children have better treatment outcomes than adults for most forms of TB, but they present different pharmacologic responses to drugs and typically require higher mg/kg doses, especially if very young [9–11]. HIV-infected persons experience complicated drug–drug interactions (DDIs) and worse TB treatment outcomes than HIV-uninfected persons and have 2–3 times greater likelihood of TB-related mortality [12]. In March 2018, the World Health Organization (WHO) held a technical consultation focused on advancing clinical trial design for more successful development of new TB treatments [13], including enrollment of key populations that may be currently underrepresented in clinical trials. Although many such populations exist, including migrants, prisoners, homeless people, and healthcare workers, the technical consultation discussions were concentrated on three populations and were framed around five questions (**Box 1**). This review is part of a Collection, “Advances in Clinical Trial Design for the Development of New TB Treatments: A Call for Innovation,” and highlights key aspects, barriers, and potential solutions to conducting TB therapeutic clinical trials in pregnant and lactating women, children, and HIV-infected persons [14].

**Box 1. Five questions addressed during discussions about key populations in clinical trials of TB therapeutics [13]**

1. Aside from the use of well-designed trials based on solid preclinical data conducted under the protections outlined in existing regulations, what are the biggest barriers to including key populations in clinical trials? What approaches or measures might stimulate greater inclusion of key populations in trials, including greater community engagement and awareness?
2. What would make the inclusion of key populations easier for researchers?
3. What special considerations need to be taken into account to include key populations into trials? Can they be included as an additional arm of study? A part of a larger patient group?
4. At what phase is it most appropriate to include key populations?
5. Areas where key populations are included should be prioritized based on burden. What are these priority areas, and what are the requirements for each population?

### Why is it important to include key populations in clinical trials?

After unanticipated harm occurred from in utero exposure to thalidomide and diethylstilbestrol in the 1960s and 1970s, the United States Food and Drug Administration (FDA) enacted policies to protect women research participants of reproductive age from teratogenic exposure [15]. An unintended consequence has been the uniform exclusion of pregnant women from Phase III trials of TB therapies, even for MDR and extremely drug-resistant (XDR) TB [7,8]. Exclusion has been based on concerns of legal liability as well as new or increased frequency/severity of adverse events and potential unpredictability of such events in pregnancy or the postpartum period. Ethical complexities and insufficient market interests for developing pediatric formulations and concerns of potential DDIs among antiretrovirals and TB therapies are among the factors preventing adequate trial data from being collected from child and HIV–TB-coinfected populations, particularly those with advanced immunosuppression.

Although concerns of potential harm from TB therapeutics are understandable, a scientific and ethical foundation exists for including pregnant and lactating women and other key populations in trials of TB medicines for prevention and treatment [16,17]—namely, the need for effective treatment and evidence-based answers to enable patients to make fully informed choices for themselves (and the developing fetus) based on risks and benefits of specific therapies. However, these data are rarely available [8,16–20]. Pregnant and lactating women, children, and HIV-infected persons each have unique features. Thus, assumptions made from therapeutic TB trials excluding these populations are not always applicable, and data cannot be reliably extrapolated from other populations. Without high-quality data from targeted studies, many unanswered questions remain concerning optimal TB regimens, optimal dosing of new/existing TB drugs, and their safety.

Although the landmark zidovudine trial paved the way for rigorous study of HIV antiretrovirals in pregnancy [21], this has yet to translate to the TB arena. TB treatment in pregnancy and lactation is mostly based on case reports and small case series [6,7,22]. As a result, medications, including those for TB, are often prescribed in pregnancy without the knowledge required to achieve appropriate doses for optimal therapeutic effect [23,24], and WHO and Centers for Disease Control and Prevention (CDC) recommend conflicting treatment guidelines for drug-susceptible TB (i.e., 6-month regimen, including pyrazinamide versus 9-month regimen, excluding pyrazinamide, respectively) [25,26]. Overall, uncertainty persists concerning optimal drug selection, safety, and timing of TB treatment initiation and whether safety signals differ by trimester.

In pediatrics, off-label drug use is a common practice and is largely based on adult studies without rigorously conducted pharmacokinetics (PK), dose-finding, or formulation studies in children [27]. Children, however, are not small adults. The age-related risk of progressing to disease after TB infection and excess risk of disseminated forms of TB in children mandate the study of new therapies in this group. Additionally, it is critical to include young, small children in trials given that the effects of age and weight on PK are most pronounced and challenging to predict in this subgroup. Notably, the 2011 revised WHO dosing guidelines for first-line TB drugs in children < 12 years old were based on studies suggesting that young children require higher mg/kg doses [28]. However, the evidence supporting these dosing recommendations was limited and especially lacking in studies using high-quality drug formulations. With a wide spectrum of disease, children with paucibacillary intrathoracic TB may in fact require lower total drug exposures (lower dose and/or shorter regimen), whereas children with more severe pulmonary TB or disseminated disease (e.g., TB meningitis) may require higher doses than adults.

Regardless of age, HIV-infected persons are at highest risk of developing TB and have a high TB-related mortality. In this population, differential responses to TB treatment and preventive regimens and overlapping toxicities between HIV therapies and TB therapies are such that safety, toxicity, and DDIs cannot be predicted by modeling alone. In particular, adults and children with advanced HIV disease have more complex and unknown responses, toxicities, and DDIs than HIV-infected persons with higher CD4 T-cell counts. This subgroup is important to include in TB trials, as they may benefit from new TB therapies, but this needs to be ascertained carefully and is best done in a clinical trial setting.

Clearly, gathering evidence under rigorous scientific conditions is among the most compelling reasons for inclusion of key populations in TB drug research [16,17,23,29,30], especially because safety signals can be more readily interpreted in a clinical study setting. Controlled trials are also essential to assess specific TB treatment–associated outcomes and adverse effects. However, there are also issues of justice and access to the benefits of research participation. Inclusion in clinical trials is likely the only way for pregnant/lactating women, children, adolescents, and HIV-infected persons to access or accelerate access to new regimens and medications.

## Overview of trial design considerations for key populations

### Pregnant and lactating women

#### Overview of TB in pregnant and lactating women

In most countries, TB incidence peaks in women of reproductive age, irrespective of HIV [22]. Pregnancy is not routinely included in national/international TB registries, but worldwide, at least 216,000 TB cases are reported to occur in pregnancy annually [2]. Immune changes in pregnancy may alter the risk of disease, TB presentation, and diagnosis [4,31,32]. Complications of TB developing during pregnancy and lactation are well known and can include maternal death, preeclampsia, vaginal bleeding, and maternal death as well as prematurity, low birth weight, and fetal or infant death, particularly if TB is inadequately treated [22,33,34]. Notably, many TB drugs are categorized by the US FDA as former category C (**Table 1**), and many have undetermined placenta crossing, fetal, or lactation compatibility [6] (**Table 1**). In addition, drug absorption, distribution, metabolism, and elimination may be modified in pregnancy and lactation [35,36], and increased clearance of some drugs requires dose modification, particularly in the third trimester [37]. Lastly, there is often a significant time gap between licensure of medicines and pregnancy-specific data being obtained. HIV antiretrovirals, which have more data in pregnancy, still had a median gap of 6 years from licensure to access [38].

**Table 1:** FDA/WHO pregnancy classification and select maternal–fetal and reproductive toxicity characteristics of drugs used to treat TB.

Drug Name	FDA^a^	WHO Grouping^b^	Crosses Placenta (Cord: Maternal Ratio)	Fetal Toxicity	Breastfeeding Compatible^b^	Teratogenic in Reproductive Toxicity Studies	Additional Concerns in Pregnancy and Postpartum
Isoniazid	C	1	Y	CNS defects	Yes (<5%)	No	Possible increased hepatotoxicity
Rifampin	C	1	Y	Hemorrhage	Yes (minimal passage, approximately 0.05% to <5%)	Yes	Possible postpartum hemorrhage; interacts with NNRTIs, PIs, decreases efficacy of hormonal contraceptives
Ethambutol	C	1/C	Yes	Jaundice	UD (minimal passage, <5%)	Yes (low incidence)	–
Pyrazinamide	C	1/C	Unknown	Jaundice	UD (excreted in breast milk)	UD	Differential recommendation between US CDC and WHO for use in TB treatment in pregnancy
Rifabutin	B	–	UD	–	UD	No	Possible postpartum hemorrhage; interacts with NNRTIs, PIs, decreases efficacy of hormonal contraceptives
Rifapentine	C	–	UD	–	UD	Yes	Possible postpartum hemorrhage; interacts with NNRTIs, PIs, decreases efficacy of hormonal contraceptives
Aminoglycosides							
Capreomycin	C	Not A–C	Yes	–	UD	Yes	–
Streptomycin	D	C	Yes	Ototoxicity, thrush, diarrhea	Yes (minimal passage)	No	–
Kanamycin	D	Not A–C	Yes	Ototoxicity	Yes (minimal passage)	No	–
Amikacin	D	C	Yes	Ototoxicity	UD	UD	–
Levofloxacin	C	A	Yes	Possible bone	Yes	No	–
Moxifloxacin	C	A	Yes	Possible bone	UD	No	–
Gatifloxacin	C	Not A–C	UD	Possible bone	UD	No	–
Ethionamide/prothionamide	C	C	UD	Developmental anomalies	UD	Yes	Developmental abnormalities in human case series
P-aminosalicylic acid	C	C	UD	Diarrhea	No	No	–
Cycloserine	C	B	UD	–	Yes	UD	Congenital sideroblastic anemia
Terizidone	–	B	UD	–	Yes	UD	–
Thioacetazone	–	Not A–C	UD	–	UD	UD	–
Clofazimine	C	B	UD	Reversible skin pigmentation	UD	No	–
Clarithromycin	C	Not A–C	Yes (0.15)	–	UD	No	–
Amoxicillin-clavulanic acid	B	Not A–C	Yes (0.56)	Necrotizing enterocolitis, transaminitis	UD	No	–
Linezolid	C	A	UD	–	UD	No	Case report of reduced PK in pregnancy
Imipenem/meropenem	C	C	UD	–	UD	No	–
High-dose isoniazid	C	Not A–C	Yes (0.73)	CNS defects	UD	No	Possible hepatotoxicity
Bedaquiline	B	A	UD	–	UD	No	Drug accumulation in tissues
Delamanid	Not approved^c^	C	UD	–	UD	Yes	Embryofetal toxicity at maternally toxic doses in rabbits; breast milk concentration 4× higher than blood in rats
Pretomanid	Not approved	–	UD	–	UD	UD	
Sutezolid	Not approved	–	UD	–	UD	UD	

Table adapted from [6].

^a^ The former FDA categories were defined as follows: category A: adequate and well-controlled studies have failed to demonstrate a risk to the fetus in the first trimester of pregnancy (and there is no evidence of risk in later trimesters); category B: animal reproduction studies have failed to demonstrate a risk to the fetus, and there are no adequate and well-controlled studies in pregnant women; category C: animal reproduction studies have shown an adverse effect on the fetus, and there are no adequate and well-controlled studies in humans, but potential benefits may warrant use of the drug in pregnant women despite potential risks; category D: there is positive evidence of human fetal risk based on adverse reaction data from investigational or marketing experience or studies in humans, but potential benefits may warrant use of the drug in pregnant women despite potential risks; category X: studies in animals or humans have demonstrated fetal abnormalities, and/or there is positive evidence of human fetal risk based on adverse reaction data from investigational or marketing experience, and the risks involved in use of the drug in pregnant women clearly outweigh potential benefits. The US FDA now uses narrative summaries to communicate what information is known and not known for individual drugs. However, the former risk categorization is still felt to be useful and has been used in this table. https://www.fda.gov/about-fda/economic-impact-analyses-fda-regulations/summary-content-and-format-labeling-human-prescription-drug-and-biological-products-requirements.

Additional information about each drug can be found at https://www.accessdata.fda.gov/scripts/cder/daf/index.cfm.

^b^ Information on breast milk transfer of TB drugs is collated on LactMed, the National Library of Medicine searchable database of drugs to which breastfeeding mothers may be exposed. https://toxnet.nlm.nih.gov/newtoxnet/lactmed.htm.

^c^ Approved by European Medicine Association and other non-FDA agencies outside the US.

Abbreviations: CDC, Centers for Disease Control and Prevention; CNS, central nervous system; FDA, Food and Drug Administration; NNRTI, nonnucleoside reverse transcriptase inhibitor; PI, protease inhibitor; PK, pharmacokinetics; UD, undetermined; WHO, World Health Organization

#### TB trial design considerations and recommendations for pregnant and lactating women

In 2018, the US FDA and the US Federal Task Force on Research Specific to Pregnant Women and Lactating Women (PREGLAC) issued separate documents to accelerate inclusion of pregnant and lactating women in clinical trials. The FDA draft guidance [23] outlines prerequisites for “reasonable” and “ethically justifiable” inclusion of pregnant women in premarketing studies (i.e., “adequate” preclinical data plus the potential to provide unique clinical benefit to the woman or fetus) and postmarketing studies (i.e., “adequate” nonclinical data plus established safety in nonpregnant women and no alternate means to extrapolate efficacy and/or assess safety). Generally, Phase I and II trials should be conducted in nonpregnant women of reproductive age, and inclusion of pregnant women should be considered in Phase III or IV trials based on clear risks and benefits assessment. Critical trial components include PK data with minimum requirements (i.e., gestational age at enrollment, gestational timing/duration of drug exposure, and pregnancy outcomes [adverse maternal, fetal, and neonatal events]), obstetrical care meeting recognized standards for pregnant women on trial, and follow-up safety data among infants of mothers with investigational drug exposure. The FDA also provides guidance regarding evaluation of systemic drug exposure to fetus/newborn, women who become pregnant on study, obtaining adequate nonclinical reproductive and developmental toxicology data, identifying trial populations standing to benefit most while minimizing risk, gestational timing of investigational drug exposure relative to fetal development, and appropriate control populations. In its report, PREGLAC highlighted 15 recommendations to encourage research on therapies during pregnancy and lactation, the majority of these being of particular relevance to TB therapeutics [18].

An international group of experts has also issued recommendations with particular reference to TB treatment trials: pregnant and lactating women should be eligible for Phase III MDR TB trials unless a compelling reason for exclusion exists, drug companies should be encouraged to complete reproductive toxicity studies of TB drugs before beginning Phase III studies, trials of shortened treatment regimens for latent TB infection (LTBI) should be designed to improve completion rates and reduce risk of progression in pregnancy and lactation, targeted PK studies should be nested in all TB studies when evidence is lacking, and a TB pregnancy registry should be established to accumulate data on maternal–infant outcomes [6]. These were discussed at the March 2018 WHO technical consultation discussions, and the following propositions were made.

#### Trial designs for active TB disease in pregnant and lactating women

Inclusion in Phase III trials is likely the only way to access more optimal regimens/newer agents and generally the only way to obtain safety, PK, and outcome data in this population, as postmarketing studies are not prioritized for funding or by regulatory bodies. In this respect, because MDR TB has significant morbidity and mortality and because many MDR TB drugs are associated with substantial intolerance and adverse effects, it is reasonable to consider inclusion of pregnant and lactating women in Phase III MDR TB treatment trials when there is no teratogenicity signal from reproductive toxicity. However, to our knowledge, no Phase III trial of MDR TB treatment has included pregnant women to date. To counter the automatic exclusion of pregnant women that currently pervades the TB trial landscape, early discussion among trialists, pharmaceutical companies, maternal–child experts, ethicists, and regulatory bodies are needed to address risks, benefits, and compelling rationale for inclusion [7].

Another important approach is to capture pregnancy outcomes among women who become pregnant while participating in a therapeutic trial. Current practice is to discontinue study drugs at the time pregnancy is identified and define the participant as “unassessable.” Instead, newly pregnant participants should be reconsented, offering the option to continue the study drug unless teratogenicity is known or suspected. All current information concerning the drug/regimen during pregnancy should be reviewed and communicated, including any shifts in risk–benefit balance, and carefully described to the patient. Examples of such secondary consent forms have been developed and are already used in some clinical trials [4]. Furthermore, support and mandates to standardize systematic data collection and reporting to a global pregnancy TB treatment registry is urgently needed. Similar to the HIV antiretroviral therapy (ART) registry, data from pregnancy, delivery, and infancy until age 6 months should be mandated [39, 40]. Whether from trials or registries, collecting PK and outcome data among pregnant women will be invaluable and can be pooled for analysis once sufficient data have accumulated. Novel physiologically based PK and pharmacodynamics (PD) modeling can also be applied to estimate drug dosing in pregnancy, but prediction of safety and toxicity profiles still requires trial data [41].

The postmarketing opportunistic PK model illustrated by International Maternal Pediatric Adolescent AIDS Clinical Trials Network (IMPAACT) P1026s [42] is another approach to advance the evidence base (**Table 2**). This protocol is enrolling pregnant and lactating women to assess the safety and PK of first- and second-line TB drugs routinely used in clinical practice as regimens evolve [43]. Assessments are made by pregnancy trimester, at delivery, and postpartum, with careful monitoring/ascertainment of maternal, fetal, and infant outcomes. PK of multiple TB drugs are captured in maternal plasma by pregnancy stage and from cord blood, breast milk, and infant samples along with relevant maternal–fetal–infant safety and clinical outcomes. This model also allows for study of DDIs between TB drugs and both antiretrovirals and postpartum contraceptives [44,45].

**Table 2:** Ongoing and planned clinical trials in pregnant and lactating women (as of December 2018).

Study/Trial Number	Funding/Sponsor	Phase	TB Type	Purpose	Design	Regimen	Study Population	Location	Status
LTBI									
IMPAACT P2001/NCT02651259	NIH NIAID, NICHD	I/II	LTBI	PK, tolerability, and safety of 3HP for LTBI	Open-label, non-randomized trial	12 once-weekly doses of P and H (3HP)	Pregnant (≥14 weeks GA)/lactating women (18 years+), **HIV+ (any CD4, compatible ARV)/**HIV−, with LTBI or known recent pulmonary TB exposure	Haiti, Kenya, Malawi, Thailand, Zimbabwe	Fully accrued/ results expected early 2020
IMPAACT P1078/NCT01494038	NIH NIAID, NICHD	IV	LTBI	Safety of antepartum versus postpartum-initiated IPT for TB prevention in HIV+ pregnant women in high-TB-burden settings	Randomized, double-blind, placebo-controlled trial	Immediate H (entry through week 28), then placebo through week 40 postpartum versus placebo (entry through week 12 postpartum), then H through week 40 postpartum	Pregnant (≥14 weeks GA)/lactating women (13 years+), **HIV+ (any CD4, any ARV**) without active TB	Botswana, Haiti, India, South Africa, Tanzania, Thailand, Uganda, Zimbabwe	Completed/ primary results presented CROI 2018 [49]
IMPAACT CS 5021	NIH NIAID, NICHD	IV	LTBI	Safety, tolerability, optimal timing, and PK of 1HP versus 3HP in pregnant and postpartum women	Open-label, randomized, 4-arm factorial design trial	1HP versus 3HP in HIV-infected pregnant and postpartum women	Recently exposed or LTBI+, **HIV+ (any CD4, compatible ARV)** pregnant (≥24 weeks GA) women; subset of HIV− for PK and safety under consideration	Multisite international	Planned
DS TB									
Tshepiso	NIH NICHD	IV	DS	PK of first-line TB drugs	Open-label, nonrandomized trial	First-line TB drugs with and without ARVs	**HIV+ (any CD4, any ARV)**/HIV− pregnant and postpartum/lactating women	South Africa	Completed. Some results published [41,44,45]
PK of first-line TB drugs in pregnancy	NIH NICHD	IV	DS	PK of first-line TB drugs	Open-label, nonrandomized trial	First-line TB drugs with and without ARVs	**HIV+ (any CD4, any ARV**)/HIV− pregnant and postpartum/lactating women	India	Ongoing
DR TB									
VirTUAL/NCT03923231	EDCTP		DS/DR	PK/PD modeling to predict doses for pregnant women, lactating women, and children	PK studies and modeled data	First- and second-line TB drugs with and without ARVs	**HIV+**/HIV− pregnant (≥20 weeks GA) and lactating women on first-line TB treatment or second-line MDR TB treatment. NCT03923231 assessing atazanavir/ritonavir with rifampin, specifically	South Africa, Uganda, United Kingdom, and Italy	Ongoing
ACTG 5300B IMPAACT2003B/NCT03568383	NIH NIAID, NICHD	III	DR	Efficacy and safety of De versus IPT for MDR TB prevention in high-risk household contacts (HIV+, non-HIV immunosuppression, LTBI, and children <5 years)	Open-label, randomized trial	De ×26 weeks versus H ×26 weeks	Children and adult household contacts of MDR TB case, **HIV+ (any CD4, any ARV**)/HIV−, possible opportunistic substudy of PK among women who become pregnant during study drug intake	27 sites on 3 continents	Accrual expected to start mid-2019. Pregnancy study under consideration
BDQ in pregnancy	South Africa MRC	IV	DR	PK of BDQ in pregnancy	Open-label, nonrandomized trial	BDQ in optimized regimen	**HIV+** (**any CD4, compatible ARV)**/HIV− pregnant and postpartum women on MDR TB treatment	South Africa	Ongoing
IMPAACT P1026s/NCT00042289	NIH NIAID, NICHD	IV	DS/DR	PK of ARVs and first- and second-line TB drugs (including BDQ and De) in pregnant women and their infants and ARVs in postpartum before/after initiation of hormonal contraceptives	Open-label, nonrandomized trial	ARVs without TB drugs; ARVs with TB drugs; no ARVs with TB drugs; +/− ARVs with second-line TB drugs; ARVs with postpartum hormonal contraceptives	**HIV+ (any CD4, compatible ARV)**/HIV− pregnant (≥20 weeks GA) and postpartum/lactating women on first-line TB treatment or second-line MDR TB treatment	US and international sites (TB mostly from South Africa)	Accrual expected mid-2019/ results expected 2025
IMPAACT 2026	NIH NIAID, NICHD	IV	DS/DR	PK of first- and second-line TB drugs in pregnant women with and without HIV	Open-label, nonrandomized trial	ARVs, contraception, and TB-related drugs during and after pregnancy	HIV−**/HIV+ (any CD4, compatible ARV)**, pregnant (≥20 weeks GA) and postpartum/lactating women on first-line TB treatment or second-line MDR TB treatment	TBD	Concept sheet in development

IMPAACT trial protocols can be found at https://impaactnetwork.org/studies/index.asp; NCT is the https://clinicaltrials.gov/ identification number; trials including HIV-infected (HIV+) are demarcated using bolded “HIV+” in the Study Population column.

Abbreviations: 1HP, 1 month of daily H and P; 3HP, 3 months of weekly H and P; ACTG, AIDS Clinical Trials Group; ARV, antiretroviral; BDQ, bedaquiline; CROI, Conference on Retroviruses and Opportunistic Infections; De, delaminid; DS, drug-sensitive; DR, drug-resistant; EDCTP, European & Developing Countries Clinical Trials Partnership; GA, gestational age; H, isoniazid; HIV, human immunodeficiency virus; IMPAACT, International Maternal Pediatric Adolescent AIDS Clinical Trials Network; IPT, isoniazid preventive therapy; LTBI, latent TB infection; MDR, multidrug-resistant; MRC, Medical Research Council; NIH, National Institutes of Health; NIAID, National Institute of Allergy and Infectious Diseases; NICHD, Eunice Kennedy Shriver National Institute of Child Health and Human Development; P, rifapentine; PD, pharmacodynamics; PK, pharmacokinetics; TB, tuberculosis; TBD, to be determined

#### Trial designs for TB preventive therapy in pregnant and lactating women

Despite the large burden of LTBI and risk of progression to active TB, pregnant women have been systematically excluded from the >12 Phase III and postmarketing clinical studies of TB preventive therapy [6,46]. Data from nonpregnant individuals and small observational studies have informed the guidance for isoniazid preventive therapy (IPT) in pregnancy [47,48]. The first randomized placebo-controlled trial to assess safety and optimal timing of IPT in HIV-infected pregnant women in high-TB-burden settings (IMPAACT P1078) was recently completed (**Table 2**) [49]. The relative risks and benefits of immediate antepartum versus deferred postpartum IPT initiation was assessed and included careful monthly monitoring of maternal, fetal, pregnancy, and infant outcomes. No differences in maternal safety outcomes, maternal–infant TB, or infant safety outcomes were found between arms, but an increase in composite adverse pregnancy outcomes was observed in the immediate IPT arm. Shorter-course, efficacious TB preventive therapy regimens have been studied in nonpregnant adults [50,51]. With greater advocacy and effort on behalf of groups focused on high-quality data for pregnant women, postmarketing trials assessing shorter LTBI regimens are also now underway or in development for pregnant women (**Table 2**). These include IMPAACT P2001 (PK and safety of 3 months of weekly isoniazid and rifapentine [3HP]) and IMPAACT Concept 5021 (safety, tolerability, optimal timing, and PK of 3HP versus 1 month of daily isoniazid and rifapentine [1HP]).

The IMPAACT network serves as an excellent example of how a group focused on therapeutics in pregnant women can make major strides to close the evidence gap (**Table 2**). Establishing a global TB registry and inclusion of pregnant women into relevant Phase III TB trials should be the next step. TB therapeutic protocols under development should be reviewed by experts in the care of TB in pregnant women, maternal–fetal medicine specialists, regulatory authorities, and bioethicists who can further comment on the risks and benefits of including pregnant women during the trial planning stage.

### Children

#### Overview of TB in children

Globally, approximately 10% of TB cases occur among children (0–14 years) annually. Of the estimated 1,000,000 cases in 2017, only 360,000 were notified to WHO, yet children < 5 years old are particularly vulnerable, accounting for >50% of child TB cases and approximately 80% of child TB-related deaths [1]. In contrast to the situation in adults, children display a wide spectrum of TB disease phenotypes ranging from nonsevere, often paucibacillary pulmonary/intrathoracic TB (usually uncomplicated lymph node disease) to severe disseminated TB and TB meningitis, a major cause of TB-related morbidity and mortality in children [52]. Paucibacillary intrathoracic TB (minimal or nonsevere TB) is more prevalent overall, and TB treatment outcomes are generally good for drug-sensitive (DS) and drug-resistant (DR) TB (provided treatment is initiated early), even when considerably lower doses of antituberculosis drugs were used for DS TB [53]. However, risk of progression from infection to active TB disease varies substantially by age and with HIV infection. PK also varies because of effects related to child age and size. Young children, particularly <2 years old, are at much higher risk of developing TB and severe disease forms [54] and typically require higher mg/kg doses of most TB drugs to reach adult therapeutic targets. Finally, TB diagnosis and treatment response monitoring rely on clinical, more subjective measures in at least 60% of children, as young children cannot spontaneously produce sputum for examination, and paucibacillary disease (sputum smear negative) is diagnosed by culture, the current diagnostic gold standard, in only 30%–40% of cases [55].

#### TB trial design considerations and recommendations for children

With concerted effort and advocacy along with academic and government funding and recognition from regulatory agencies, the pediatric TB trial landscape has substantially improved, as evidenced by the number of ongoing and planned studies of treatment for the diverse forms of TB in children (**Table 3**). The ways in which pediatric and adult TB differ inform the type of pediatric TB drug trials needed and their key design considerations. If children are to be included in adult trials, different inclusion and exclusion criteria may be needed, and definitions used to determine study endpoints (e.g., unfavorable outcome) require careful consideration because of differing clinical features and diagnostic challenges of TB in children compared with adults. Diagnosis, treatment response monitoring, and characterization of treatment outcome in children often depend on clinical measures that are relatively imprecise compared with the diagnostic standard used in adults. Limited availability of pediatric-friendly formulations also poses a barrier to enrollment of younger children. Large Phase III clinical trials may not be feasible or always needed for children, yet timely PK and safety data in children, especially in young and HIV-infected children, is critical to inform policy guidance on new therapies deemed to be safe and efficacious in adolescent and adult populations. Modified study designs should be explored to accelerate implementation of PK and safety studies in children while ensuring the validity of the trial results and the safety of all child participants. Unlike younger children, adolescents (typically ≥10 years old) have TB disease characteristics similar to adults, including frequent cavitating disease. Adolescents should therefore be routinely considered for inclusion in adult Phase IIb and III trials. However, similar to pregnant and lactating women, legal requirements for child participation in clinical trials are often barriers (perceived or real) and vary by country. When feasible and justified through appropriate consultation, the inclusion of children should be carefully considered and supported early during protocol development. Summaries of considerations for the types of trials needed for children, including practical and ethical considerations regarding inclusion of children in TB trials, can be found elsewhere [5, 56]. Highlights and considerations discussed at the WHO technical consultation are discussed below based on updated information.

**Table 3:** Ongoing and planned TB clinical trials in children (as of December 2018).

Study/Trial Number	Funding/ Sponsor	Phase	TB Type	Purpose	Design	Regimen	Study Population	Location	Status
LTBI									
TBTC Study 35/NCT03730181	TBTC, CDC	I/II	LTBI	Optimal dose, PK, and safety of 3HP for LTBI in HIV+/− children	Open-label PK and safety trial of P and H coformulation	P in fixed dose combination + H + P single formulation	Infants and children (0–12 years old), **HIV+**/−, modified age de-escalation, population PK modeling	South Africa	Accrual expected to start 2019
IMPAACT CS 5019	NIH NIAID, NICHD	I/II	LTBI	PK, safety, and tolerability of 1HP in HIV-infected and uninfected children with exposure to DS TB	Multicenter, open-label dose-finding and safety study	1HP with integrase inhibitors in HIV-infected children	Infants, children, and adolescents <12 years old, **HIV+**/−	Multisite international	Planned
iTIPS/NCT02613169	Thrasher Research Fund	II	LTBI	Efficacy of INH to prevent MTB in HIV-exposed uninfected infants	Randomized control trial	Daily H ×12 months versus no H	Infants (6 weeks), HIV-exposed	Kenya	Fully accrued
P4v9 Trial/NCT00170209	Canadian Institutes of Health Research	III	LTBI	Efficacy, safety, and tolerability of R and H for LTBI	Multicenter, open-label, randomized positive-controlled trial	R ×4 months versus H ×9 months	Children and adolescents (<18 years), children with LTBI at high risk of TB	Canada, Australia, Benin, Ghana, Guinea, Indonesia	Fully accrued
TB-CHAMP/ISRCTN92634082	Joint Global Health Trials Scheme, South African MRC	III	LTBI	Efficacy of Le for MDR TB prevention in HIV+/− child household contacts	Multicenter, cluster randomized, double-blind, placebo-controlled, superiority trial	Daily Le ×6 months versus placebo	Infants and children (0 to <5 years old), **HIV+**/HIV−, household randomization, IGRA+/−	South Africa	Enrolling
V-QUIN/ACTRN12616000215426	Australian National Health and MRC	III	LTBI	Efficacy of Le for MDR TB prevention in adult and adolescent household contacts	Multicenter, randomized, double-blind placebo-controlled, superiority trial	Daily Le ×6 months versus placebo	Adolescents and adults, **HIV+**/−, household randomization, TST+	Vietnam	Enrolling
A5300B I2003B/NCT03568383 (PHOENIx)	NIH NIAID, NICHD	III	LTBI	Efficacy and safety of De versus standard-dose H for MDR TB prevention in high-risk household contacts	Multicenter, open-label, randomized superiority trial	Daily De ×26 weeks versus daily H + vitamin B6 ×26 weeks	Adults, adolescents, children, infants, **HIV+**/−, household randomization	Botswana, Brazil Peru, India, Philippines, Haiti South Africa, Thailand, Kenya	Planned to open 2019
DS TB									
DAtiC/NICHD069175 NCT01637558	NIH NICHD	I	DS	PK of first-line TB drugs using 2010 WHO guidelines across pediatric populations	Intensive PK sampling of HRZE	ATT no ART, ATT + LPV/r-based ART; no ATT on LPV/r-based ART; ATT + NVP-based ART	Children and infants (0–12 years), **HIV+**/HIV−, malnutrition, drug–drug interactions, population PK modeling	South Africa, Malawi	Fully accrued
OptiRif Kids	TB Alliance, Unitaid	I	DS	PK, safety, and dose optimization of R for TB treatment in children and infants	Intensive PK sampling	High-dose R	Infants and children (0–12 years old), HIV−	South Africa	Fully accrued
Treat infant TB	TB Alliance, Unitaid	I	DS	PK and safety of first-line TB drugs using 2010 WHO dosing in infants	Intensive PK sampling, first-line TB drugs, single-drug formulation	Standard-dose HRZ	Infants <12 months, **HIV+**/−	South Africa	Fully accrued
IMPAACT P1101/NCT01751568	NIH NIAID, NICHD	I/II	DS	Safety and tolerability of raltegravir with R-containing TB regimen in infants and children	Open-label, dose-finding, safety, tolerance, and PK study of raltegravir	Chewable raltegravir tablets + 2NRTIs + R-containing TB regimen	**HIV+**/TB-coinfected children (≥4 weeks to <12 years old), received ≥1 week and ≤20 weeks of R-based TB therapy prior to ARV initiation	South Africa	Enrolling
HIVPED001/NCT02348177	AFD, MSF, AECID Spain; SDC	I/II	DS	Safety, tolerability, and virological effect of “superboosting” in HIV–TB-coinfected infants and children	Multicenter, open-label, nonrandomized, noninferiority PK study	Super-boosted LPV/r (1:1) + R versus standard-boosted LPV/r (4:1) without R	Children, infants, (>42 weeks old) **HIV+**, clinical TB diagnosis	South Africa, Thailand, France	Fully accrued
TBM-KIDS/NCT02958709	NIH NICHD	II	DS	Efficacy, PK, and safety of high-dose R +/− Le for TB meningitis in children	Open-label, randomized trial	High-dose R + EHZ ×2 months/10HR versus high-dose R + LeHZ ×2 months/10HR versus standard of care (2REHZ/10HR)	Children and infants (6 months–12 years), **HIV+**/−, intensive PK, population PK modeling	India, Malawi	Enrolling
SHINE study/ISRCTN63579542	Joint Global Health Trials Scheme	III	DS	Efficacy and safety of shortened first-line TB regimen using 2010 WHO-recommended doses for minimal TB in children	Open-label, randomized, noninferiority trial	2HRZ(E)/2HR versus 2HRZ(E)/4HR	Children, adolescents, and infants (0–16 years old), **HIV+**/HIV−, nested PK studies, drug–drug interactions	India, Uganda, Zambia, South Africa	Fully accrued
SURE-TBM/ISRCTN40829906	MRC, DFID, NIHR, Wellcome Trust	III	DS	Efficacy and safety of high-dose R, Le, and H with Z for shortening TB meningitis treatment to 6 months	Open-label, randomized, noninferiority trial	Higher dose (6RLeHZ) versus WHO standard of care regimen (2HRZE/10HR)	Infants, children, and adolescents (28 days–15 years old), **HIV+**/−	Vietnam, India, Uganda, Zambia, Zimbabwe	Planned
PK-PTB HIV01/NCT01687504 NCT01699633 NCT01704144	NIH NICHD	IV	DS	PK and safety of WHO- recommended increased dosages of first-line TB drugs in children with TB and HIV/TB coinfection	Open-label, steady-state PK study of first-line TB drugs and ARVs		Children, infants (3–14 years old), **HIV+**/−, drug–drug interactions	Ghana	Fully accrued
Rifabutin PK trial	ICMR, NACO	IV	DS	PK and safety of rifabutin	PK and safety	Rifabutin	Adults, children, HIV−	India	Planned
DR TB									
MDR-PK 1	NIH NICHD	I/II	DR	PK, safety of second-line drugs for MDR TB, particularly Mo, Le, and Li	Semi-intensive PK sampling, model-based analysis	Ethionamide, Le, ofloxacin, Mo, high-dose H, PZA, terizidone, PAS	Children, infants, adolescents (<18 years), **HIV+**/HIV−, drug–drug interactions	South Africa	Fully accrued
MDR-PK2	NIH NICHD	I/II	DR	PK, safety of second-line drugs for MDR TB, particularly Mo, Le, and Li	Semi-intensive PK sampling, model-based analysis	Li, Mo, Le, clofamizine, BDQ	Children, infants, adolescents (<18 years old), **HIV+**/−, drug–drug interactions	South Africa	Fully accrued
IMPAACT P1108 NCT02906007	NIH NIAID, NICHD	I/II	DR	PK, safety, and tolerability of BDQ for MDR TB	Open-label, single-arm, dose-finding and safety study	BDQ ×24 weeks + routine background MDR therapy	Children, infants, adolescents (0–18 years old), **HIV+**/−, population PK modeling, modified age de-escalation	South Africa, India, Haiti	Enrolling
232 and 233 NCT01856634 NCT01859923	Otsuka	I/II	DR	PK, safety, tolerability, and efficacy of De + MDR TB therapy in HIV−	Open-label, single-arm dose-finding trial	Multiple doses of De ×6 months + OBR	Children, infants, adolescents (0–17 years old), HIV−, population PK modeling, age de-escalation	Philippines, South Africa	Fully accrued
IMPAACT 2005 NCT03141060	NIH NIAID, NICHD	I/II	DR	PK, safety, tolerability of De + OBR for MDR TB in HIV+/− children	Multisite, open-label, single-arm dose-finding trial	De ×6 months + OBR	Children, infants, adolescents (<18 years old), **HIV+**/−, population PK modeling	Botswana, India, South Africa, Tanzania	Enrolling
Janssen C211 NCT02354014	Janssen	II	DR	PK, safety, tolerability of BDQ + OBR for MDR TB	Multicenter, open-label, single-arm, dose-finding and safety trial	BDQ ×24 weeks + OBR	Children, infants, adolescents (0–18 years old) HIV−, age de-escalation	Russian Federation, South Africa, Philippines	Enrolling
IMPAACT 2020 “Smart Kids”	NIH NIAID, NICHD	II	DR	Safety, efficacy of oral 6-month regimens for RR/ MDR/pre-XDR/XDR TB	Multicenter, open-label, randomized trial	Oral 6-month regimen BDQ, De, Li, Le (clofazimine for FQ resistant)	Infants, children, adolescents (0–15 years old), **HIV+**/−	Multisite	Planned
IMPAACT P1106 NCT02383849	NIH NIAID, NICHD	IV	DS/DR	PK and safety of R and H with NVP or LPV/r in low-birth-weight infants	Open-label, nonrandomized PK study of ARVs and TB medicines	NVP versus NVP + H versus NVP + H + R versus H alone or H + R versus LPV/r + 2NRTIs +/− H versus LPV/r + 2NRTIs + R +/− H	Infants (7–14 days old), **HIV+**/−, low birth weight, premature	South Africa	Enrolling

IMPAACT trial protocols can be found at https://impaactnetwork.org/research-areas/tuberculosis.htm; NCT is the https://clinicaltrials.gov identification number; trials including HIV-infected (HIV+) persons are demarcated using bolded “HIV+” in the Study Population column.

Abbreviations: 1HP, 1 month of daily isoniazid and rifapentine; 3HP, 3 months of weekly isoniazid and rifapentine; AECID, Agencia Española de Cooperación Internacional para el Desarrollo (Spanish Agency for International Development Corporation); ART, antiretroviral therapy; ARV, antiretroviral; AFD, French Development Agency; ATT, antituberculosis therapy; BDQ, bedaquiline; CDC, Centers for Disease Control and Prevention; De, delamanid; DFID, British Department for International Development; DS, drug-sensitive; DR, drug-resistant; E, ethambutol; FQ, fluoroquinolone; H, isoniazid; HIV, human immunodeficiency virus; ICMR, Indian Council of Medical Research; IGRA, interferon gamma release assay; IMPAACT, International Maternal Pediatric Adolescent AIDS Clinical Trials Network; INH, isoniazid; Le, levofloxacin; Li, linezolid; LPV, lopinavir; LTBI, latent TB infection; Mo, moxifloxacin; MDR, multidrug-resistant; MRC, Medical Research Council; MSF, Médecins Sans Frontières; NACO, National AIDS Control Organization; NIAID, National Institute of Allergy and Infectious Diseases; NICHD, Eunice Kennedy Shriver National Institute of Child Health and Human Development; NIH, National Institutes of Health; NIHR, National Institute for Health Research; NRTI, nucleoside reverse transcriptase inhibitor; NVP, nevirapine; OBR, optimized background regimen; P, rifapentine; PAS, P-aminosalicylic acid; PK, pharmacokinetics; PZA, pyrazinamide; R, rifampin; RR, rifampicin-resistant; SDC, Swiss Agency for Development and Cooperation; TB, tuberculosis; TBTC, Tuberculosis Trials Consortium; TST+, tuberculin skin test positive; WHO, World Health Organization; XDR, extremely drug-resistant; Z, pyrazinamide

#### Trial designs for active TB disease in children

Considering scenarios in which disease progression and/or response to an intervention are expected to differ among adults and children, the classical approach is to conduct PK studies in children to establish appropriate dosing followed by safety and efficacy trials. For example, because children often develop less severe, paucibacillary TB, it is plausible that children would respond equally well (i.e., treatment would have at least equal efficacy) to shorter, less intense, and less complex regimens than adults while potentially improving their tolerability, safety, acceptability, and adherence. Identifying such regimens would require an efficacy study in children, as regimens that could be effective in children may be rejected in adult trials. Based on these assumptions, the currently ongoing Shorter Treatment for Minimal TB in Children (SHINE) trial (ISRCTN63579542) investigates whether a shorter 4-month regimen can be used for children with less severe disease than the standard 6-month adult regimen (**Table 3**). Other examples include the treatment of LTBI (discussed below) and TB meningitis. TBM Kids (NCT 02958709) is the only currently open trial to assess the treatment of TB meningitis, which especially affects very young children.

In contrast, when considering scenarios in which children and adults are expected to have similar disease progression, response to an intervention, and exposure response, then it is logical to conduct PK studies to achieve drug exposures similar to adults, followed by safety trials at the proper dose. For individual TB medications, it is reasonable to assume that the response in children would be at least as good as in adults. Therefore, repeating formal efficacy studies for individual TB drugs in children is unnecessary. Instead, the focus should be on trials to establish PK, dose, and safety in children. Many of the trials shown in **Table 3** are such studies, including the pediatric trials of the recently approved drugs bedaquiline and delamanid. Another example is the Opti-Rif Kids trial (South African trial identifier 27-0117-5411), which aims to characterize rifampin doses among children 0 to <12 years old that approximate exposures observed in adults receiving higher rifampin doses (≥35 mg/kg) in adult trials [57]. Both age and weight have an impact on PK in children and must be considered in the design of pediatric PK studies of TB drugs. It is especially critical to include young, small children given that the effects of age and weight are most pronounced in this subgroup. Traditionally, age-de-escalation studies have been a major feature of pediatric PK-focused Phase I/II trials whereby children have been studied in series, rather than in parallel, starting with older children and progressing to younger children. This approach, however, should be avoided if possible: it is costly and time consuming; older children may have limited ability to inform dosing and safety in the youngest children, for whom there is the most uncertainty; and regulatory agencies do not strictly require age de-escalation [5]. HIV infection and malnutrition are additional, important covariates to consider when designing pediatric trials, and these children should be included in TB therapeutic trials.

If the exposure response to an intervention is expected to differ among children and adults, then PK/PD should be conducted to establish the exposure response in children followed by safety studies. If a PD marker is unavailable to assess pharmacologic response, as is typically the case in bacteriologically unconfirmed TB (i.e., clinically diagnosed TB), then PK studies should be followed by safety and efficacy studies [56]. The traditional assumption that exposure response is similar among children and adults for all types of TB is being questioned. For example, most children with pulmonary TB are sputum smear and culture negative and therefore have different bacillary burden compared with adults with cavitating disease. Given that childhood TB may differ in disease type and severity compared with adult TB, target concentrations for treatment of many forms of childhood TB may differ from those in adults. This provides additional justification for efficacy trials in children in some instances. For example, there are no data from trials investigating regimens to prevent MDR TB in either adult or child household contacts. TB-CHAMP (ISRCTN92634082) is a Phase III cluster-randomized placebo-controlled study that is specifically powered to evaluate the efficacy of 6 months of levofloxacin versus placebo for the prevention of TB in young child household contacts (age < 5 years) of MDR TB cases. Although not powered for efficacy in children, the PHOENIx trial (A5300/I2003) plans to study adult, HIV-infected, and child contacts of MDR TB using delamanid versus isoniazid and is a good example of how key populations can be studied within a single Phase III efficacy trial (**Table 2**).

Lastly, child-friendly formulations are important to ensure accurate, acceptable, and palatable doses in young children. The development and implementation of bioequivalence studies of pediatric formulations is lengthy and should start much earlier during the drug development process. A potential temporary solution is to better understand how manipulating the adult formulation affects the PK to inform pediatric use. The TASK-002 study successfully assessed the relative bioavailability of 100-mg bedaquiline tablets suspended in water versus when administered in healthy adult volunteers to inform its use in children [58]. This does not eliminate the need for making pediatric formulations available but does improve access to much-needed medications during the timeframe following trial completion and drug registration until routine medication availability.

### HIV-infected persons

#### Overview of TB in HIV-infected persons

Worldwide, an estimated 1,040,000 TB cases and 300,000 TB deaths occurred among HIV-infected persons in 2017–86% of reported HIV-associated TB deaths occurred in sub-Saharan Africa [1]. TB is 20–30 times more likely in the context of HIV and remains the leading cause of death in this population. Adults and children with advanced HIV disease (low CD4 count) are especially vulnerable. This subgroup has a particularly high mortality rate [59] and is more likely to have disseminated TB disease and more rapid disease progression. Despite this, a 2011 review revealed that many TB trials exclude HIV-infected persons with CD4 counts < 200–350 cells/mm^3^ [60]; our review of recent [61–64], currently enrolling, and registered (clinicaltrials.gov) randomized TB trials suggests recent expansion of inclusion criteria, but HIV-infected persons with very low CD4 counts (<50–100 cells/mm^3^) remain frequently excluded (**Table 4**). Overall, clinical management of dual TB–HIV disease is complex [12,65]. As in children, smear-negative TB disease is common in the context of HIV, which poses challenges for TB diagnosis and treatment monitoring. In addition, polypharmacy arising from treatment of HIV, TB, and new/existing comorbidities may increase adverse events and impact adherence and tolerability. Drug metabolism, absorption, and toxicity profiles may be altered in HIV, making longer courses of treatment and side effects, such as neuropathy, liver injury, and rash, more likely [66,67]. Immune reconstitution inflammatory syndrome (IRIS)/paradoxical worsening, specific cytochrome interactions, poor nutritional status, and chronic inflammation further impact HIV-infected populations. As in children and pregnant women, physiologically based PK modeling can help inform TB drug dosing in the setting of HIV but cannot replace data generated from trials. In recent years, high-quality evidence has dramatically evolved the use and timing of TB treatment in relation to ART [68]—persons with advanced HIV who are diagnosed with TB are currently recommended to start ART within 2 weeks [69,70]. However, potential DDIs remain a major concern for TB treatment in HIV-infected persons, particularly between antiretrovirals, such as protease inhibitors and integrase inhibitors, and rifamycins, key TB sterilizing agents [12,65]. DDIs and adverse effects cannot always be readily identified from observations in HIV-uninfected populations. A healthy-volunteer study assessing a TB-preventive regimen (rifapentine and isoniazid) and interaction with dolutegravir (HIV antiretroviral) found significant toxicity and was terminated early, yet these effects were not observed in a larger study of HIV-infected persons [71,72]. It is important that TB trials assess the full spectrum of HIV/TB and be sufficiently powered to evaluate the impact of HIV [41,60].

**Table 4:** Ongoing and planned TB clinical trials in HIV-infected persons 12 years and older (as of December 2018).

Study/Trial Number	Funding/ Sponsor	Phase	TB Type	Purpose	Design	Regimen	Study Population	Location	Status
LTBI									
WHIP3TB/NCT02980016	USAID	III	LTBI	Efficacy of 3HP given once or annually to reduce TB	Open-label, randomized trial	Part A: 6H versus 3HP; part B: 3HP once versus annually	Adults, adolescents, children (2+ years old), **HIV+ on ART 3+ months or not eligible for ART, any CD4**	South Africa, Mozambique, Ethiopia	Enrolling
TBTC37	CDC	III	LTBI	Efficacy and safety of 6 weeks of HP daily	Open-label, randomized trial	6 weeks daily HP versus 3HP versus 4HR daily versus 4R daily	Adults and adolescents (12+ years old), **HIV+**/−, on **compatible ART, any CD4**	US, TBTC international sites TBD	In development
DS TB									
NCT03563599	Qurient	IIa	DS	Assess early bactericidal activity of Telacebec	Open-label, randomized trial	Multiple doses of Telacebec (Q203) versus Rifafour e-275 (RHZE)	Adult (18–65 years old), new treatment-naïve smear-positive DS TB, **no HIV exclusion criteria stated**	South Africa	Enrollment complete March 19, 2019
ReDEFINe/NCT02169882	Universitas Padjadjaran, USAID	IIb	DS	Dose finding for R to treat TB meningitis	Double-blind randomized trial	Standard dose versus R_(900)_ or R_(1350)_ + HEZ ×6 months	Adults (15+ years old), no pregnancy/breastfeeding, on ATT <3 days with clinical suspicion of TBM**, no HIV exclusion criteria stated**	Indonesia	In data analysis
APT/NCT02256696	FDA	IIb	DS	Mycobacterial activity of Pa824	Open-label, randomized trial	Pa824_(200)_ ×12 weeks added to HRZ	Adults (18+ years old); HIV−/**HIV+ CD4 ≥350 cells/mm**^**3**^ **and not on ART**	South Africa	Enrolling
ACTG5362 CLOFAST	NIH NIAID	IIc	DS	Dose finding for C to treat DS TB	Double-blind randomized trial	(4C_50_ versus 4C_100_ versus 4placebo) + 4HP_1200_ZE/2placebo versus 2placebo versus 2HP	Adults (18+ years old), no pregnancy/ breastfeeding, **HIV+ CD4 ≥ 100 cells/mm**^**3**^**, compatible ARV or about to start**	ACTG sites TBD	In development
NCT02836483	LegoChem Biosciences	II	DS	Early bactericidal activity, safety, and PK of oral delpazolid	Open-label, randomized trial	Multiple doses of delpazolid versus Li	Korean adults (19–70 years old) with smear-positive pulmonary TB. **No HIV exclusion criteria stated**	Korea	Enrolling
TBTC Study 31 ACTG 5349/NCT02410772	AIDS Clinical Trials Group, CDC	III	DS	Efficacy of 2 shortened rifapentine-containing regimens for pulmonary TB	Open-label, randomized, controlled clinical trial	Standard 6-month regimen versus 4-month regimen substituting P for R versus 4-month regimen substituting P for R and M for E	Children and adults (12 years+), AFB or GeneXpert-positive, documented HIV status, **if HIV+ CD4 > 100 cells/mm**^**3**^	USA, Brazil, China, Haiti, India, Kenya, Malawi, Peru, South Africa, Thailand, Uganda, Vietnam, Zimbabwe	Enrollment completed. Follow-up ongoing.
DR TB									
ACTG 5356	NIH NIAID	IIa	DR	Dose-finding for Li in all oral regimens for MDR TB	Open-label, randomized trial	Li (600 qd/1,200 qd) + Bdq200 +De200 + Le (if FQ S) or C (if FQ R)	Adults and adolescents (>12 years old); **if HIV+ CD4 ≥ 50 cells/mm**^**3**^	ACTG sites TBD	In development
TBTC Study 32, OPTI-Q NCT01918397	CDC, NIH NIAID	II	DR	Efficacy, safety, and tolerability of using Le in regimen for MDR TB	Blinded, randomized PK/PD trial	4 doses of Le + OBR	Adults (18+ years old), smear-positive/culture-positive MDR TB; **HIV**+ **included must have viral load and CD4 count within 3 months**	Peru, South Africa	Enrolling
ACTG 5343/NCT02583048	NIH NIAID	II	DR	Safety, tolerability, and PK of BDQ and De (alone and in combination) + OBR for RR/MDR TB	Open-label, randomized trial	6 months of BDQ + OBR versus De + OBR versus BDQ + De + OBR; dolutegravir + 2 NRTIs for HIV+ only	Adults (18+ years old), documented RR/MDR pulmonary TB, documented HIV status, if **HIV+**: **CD4 > 100 cells/mm**^**3**^ **and one fully active NRTI available if on ART >6 months and viral load > 500 copies/mL**	Peru, South Africa	Active, not recruiting
MDR END/NCT02619994	University Seoul, Korea	II	DR	Safety, efficacy of shortened injection-free regimen for MDR TB	Open-label, randomized controlled clinical trial	De + Le + Li + Z x 9 or 12 months versus 24 OBR	Adults 19+ years old, no FQ resistance, **no HIV exclusion criteria stated**	Korea	Enrolling
SODOCU	EDCTP	II	DR	Dose-finding study of U	Open-label dose-finding trial	3U (0 mg qd + 600 mg qd versus 1,200 mg qd versus 600 mg bid versus 800 mg bid) + 3BdqDeM	Adults	TBD	In development
SimpliciTB/NCT03338621	Global Alliance for TB Drug Development	II/III	DS/DR	Efficacy, safety, and tolerability of a new, shorter oral regimen for DS/DR TB	Open-label, partially randomized trial	DS TB: BDQPaMoZ ×4 months versus HRZE/HR ×6 months; DR TB: BdqPaMoZ ×6 months	Adults (18+ years old), new smear-positive DS/DR TB; **HIV+ criteria**: **CD >100 cells/mm**^**3**^, Karnofsky score >60%, no IV antifungal in past 90 days, and WHO clinical stage <4 disease	10 countries in Africa, Asia, Europe, and South America	Enrolling
TB PRACTECAL/NCT02589782	MSF, Global Alliance for TB Drug Development, WHO, THINK	II/III	DR	Safety (Phase II) and efficacy (Phase III) of short regimens containing B and Pa for MDR/XDR TB	Open-label, randomized trial	6 months of BdqPaLiMo, BdqPaLiC, or BdqPaLi versus local WHO SOC MDR/XDR TB regimen	Adults (18+ years old), with microbiologically confirmed TB resistant to at least R; **HIV+ included regardless of status**	Belarus, South Africa, Uzbekistan	Enrolling
NExT-5001/NCT02454205	University of Cape Town	II/III	DR	Efficacy, safety, tolerability of shortened, injection-free regimen for MDR TB	Open-label, randomized controlled clinical trial	LiBdqLeZ + E or high-dose H ×6–9 months versus conventional empiric injection-based 21–24 month regimen	Adults (18+ years old), new culture or GeneXpert-positive MDR TB; **if HIV+ CD4 > 50 cells/mm**^**3**^	South Africa	Enrolling
ACTG 5273 FIRST	NIH NIAID	III	DR	Efficacy of new regimens for H monoresistant TB	Open-label, randomized clinical trial	6H 15mg/kg RZE versus 2RZELe/2RLe	Adults, adolescents, and children; **any CD4, any ARV**	ACTG sites TBD	In development
STREAM/NCT02409290	IUATLD	III	DR	Efficacy of different regimens for MDR TB	Open-label, randomized clinical trial	Local 2011 WHO MDR TB regimen versus CEMZ ×40 weeks + H, kanamycin, prothionamide × first 16 weeks versus 40 weeks oral regimen BdqCELeZ + H and prothionamide × first 16 weeks versus 28 weeks BdqCLeZ + H and kanamycin × first 8 weeks	Adult (15+ years old), AFB or GeneXpert positive, resistant to rifampicin and isoniazid, **if HIV+: willing to start ART and CD4 > 50 cells/mm**^**3**^	Ethiopia, Georgia, India, Republic of Moldava, Mongolia South Africa, Uganda	Enrolling
Nix-TB (B-Pa-L)/NCT02333799	Global Alliance for TB Drug Development	III	DR	Safety, efficacy, tolerability, and PK of BDQ + Li ×6 months for MDR/XDR TB	Open-label trial	6–9 months of BdqPaLi	Children and adults (14+ years old), XDR TB or nonresponsive MDR TB, culture-positive, documented HIV status, **if HIV+ CD4 > 50 cells/mm**^**3**^	South Africa	All enrolled
ZeNix NC-007/NCT03086486	Global Alliance for TB Drug Development	III	DR	Safety and efficacy of various doses and treatment duration of Li + Pa + BDQ for MDR, pre-XDR, and XDR TB	Open-label, partially blinded, randomized clinical trial; even allocation across arms by HIV status and TB type	Li_(1,200)_ ×26 weeks + Pa + BDQ versus Li_(1,200)_ ×9 weeks + Pa + BDQ versus Li_(600)_ ×26 weeks + Pa + BDQ versus Li_(600)_ ×9 weeks + Pa + BDQ	Children and adults (14+ years old), documented HIV status, culture or molecular test positive and documented resistance, **if HIV+ CD4 > 100cells/mm**^**3**^	Georgia, Republic of Moldova, Russian Federation, South Africa	Enrolling
endTB/NCT02754765	UNITAID	III	DR	Evaluating newly approved oral, shortened regimens for MDR TB (FQ sensitive)	Open-label, randomized, controlled noninferiority clinical trial	BdqLiMoZ ×39 weeks BdqLiCLeZ ×39 weeks BdqDeLiLeZ ×39 weeks DeLiCLeZ ×39 weeks DeCMoZ ×39 weeks versus control (Z)	Children and adults (15+ years old) with documented pulmonary MTB resistant to R, **no HIV exclusion criteria stated**	Georgia, Kazakhstan, Kyrgyzstan, Lesotho, Peru, and South Africa	Enrolling
endTB-Q	UNITAID/MSF	III	DR	Evaluating newly approved oral, shortened regimens for MDR TB (FQ sensitive)	Open-label, randomized, controlled noninferiority clinical trial	6BdqDeLiC versus 10BdqDeLiC versus OBR	Children and adults (15+ years old) with documented pulmonary MTB resistant to R; **no HIV exclusion criteria stated**	India, Pakistan, Kazakhstan, Lesotho, Peru	In development
BEAT TB	South Africa	III	DR	Safety and efficacy of short regimen for MDR TB	Strategy trial	6BdqDeLeLi_600_C (drop Li if FQ sensitive; drop Le if FQ resistant)	Adults	South Africa	In development
DRAMATIC/NCT03828201	US Department of Defense	III	DR	Efficacy and tolerability of shortened injection-free regimen for MDR TB	Open-label, randomized controlled clinical trial	4BdqDeLe_1000_Li_1200_C versus 6BdqDeLe_1000_Li_1200_C versus 2011 WHO regimen	Adults and adolescents 12+ years old, HIV−/ **HIV+ any CD4**	TBD	In development

Data in this table obtained from clinical trials.gov and adapted from a table compiled by Michael J. Vjecha, MD, on behalf of TBTC Core Science Group.

Abbreviations: 3HP, 3 months of weekly isoniazid and rifapentine; ACTG, AIDS Clinical Trials Group; AFB, acid-fast bacilli; ART, antiretroviral therapy; ARV, antiretroviral; ATT, antituberculosis therapy; Bdq, bedaquiline; C, clofazimine; CDC, Centers for Disease Control and prevention; De, delamanid; E, ethambutol; EDCTP, European & Developing Countries Clinical Trials Partnership; FDA, Food and Drug Administration; FQ, fluoroquinolone; HIV, human immunodeficiency virus; H, isoniazid; IUATLD, International Union Against Tuberculosis and Lung Disease (The Union); Le, levofloxacin; Li, linezolid; LTBI, latent TB infection; MDR, multidrug-resistant; Mo, moxifloxacin; MSF, Médecins Sans Frontières; NIAID, National Institute of Allergy and Infectious Diseases; NIH, National Institutes of Health; NRTI, nucleoside reverse transcriptase inhibitor; OBR, optimized background regimen; P, rifapentine; Pa, pretomanid; PD, pharmacodynamics; PK, pharmacokinetics; R, rifampicin; RR, rifampicin-resistant; s, sensitive; SOC, standard of care; TB, tuberculosis; TBD, to be determined; TBTC, Tuberculosis Trials Consortium; THINK, TB&HIV Investigative Network; U, sutezolid; USAID, United States Agency for International Development; WHO, World Health Organization; XDR, extremely drug-resistant; Z, pyrazinamide

#### Trial design considerations and recommendations for TB disease and preventive therapies in HIV-infected persons

Inclusion of HIV–TB-coinfected populations in TB clinical trials poses a number of challenges. To enhance their enrollment, TB trials should be conducted, at least in part, in geographic locations where HIV and TB epidemics coincide and interact. Partnering with public-funded trials networks specializing in recruitment of HIV-infected persons can facilitate this. For example, the US CDC Tuberculosis Trials Consortium (TBTC)/AIDS Clinical Trials Group (ACTG) partnership has enhanced enrollment of HIV-infected people in the Phase III randomized trial of rifapentine-containing shortened treatment for pulmonary TB (NCT02410772). Requesting culture-confirmed disease for trial eligibility also limits enrollment of HIV–TB-coinfected persons. Sensitivity of sputum smear and culture are limited by low bacillary load of TB in the context of HIV [73]. As in young children, less stringent measures, such as clinical TB diagnosis, could be incorporated. To ensure balanced treatment assignments among various trial subgroups, randomization could be stratified by HIV status (i.e., HIV-infected versus -uninfected) or by specific eligibility criteria (i.e., culture-confirmed versus nonconfirmed). Incorporating clinical TB diagnosis as a secondary outcome measure (ideally reviewed by an expert committee blinded to treatment assignment) may also be important for interpreting results in the overall trial population and in key subgroups. Outcome rates could also be assessed by HIV infection/HIV disease status and/or ART use, as treatment outcomes in HIV–TB-coinfected patients may be highly dependent on the specifics of ART management. Consistent with HIV and TB treatment guidelines, ART should be required or expected to be initiated within 4–8 weeks of initiating TB treatment. It is important to understand whether mortality or other poor outcomes in HIV–TB-coinfected patients is related to HIV or TB. Thus, data analysis should be stratified by HIV infection/HIV disease status (i.e., HIV-uninfected, HIV-infected with high CD4 count, and HIV-infected with low CD4 count) to reduce concerns about any potential imbalances in subgroup numbers between randomized arms.

Carefully designed DDI studies are a major element of clinical research of TB therapeutics for treatment and prevention of TB in HIV-infected people, including HIV-infected adults and children [74]. DDIs may be bidirectional, and the potential impact of host genetics is difficult to predict from small PK studies alone. To facilitate enrollment of HIV-infected individuals, DDI studies should be conducted early in drug development and/or nested in major trials [41]. The Phase III randomized ACTG 5279 trial, “Short-Course Rifapentine/Isoniazid for Treatment of Latent TB in HIV-Infected Individuals” (NCT01404312)[51], is an example of a nested DDI study: the first 90 participants that were on efavirenz-based ART and randomized to the rifapentine arm entered into a semi-intensive PK study [75] and were evaluated for PK/PD and potential HIV virologic failure to confirm that efavirenz PK and ART outcomes remained adequate. As in this example, the risk to a TB trial may be lower if PK of an HIV drug is the concern, particularly for shorter periods of TB drug use. If the potential DDI involves one of the TB drugs and may affect the randomized comparison, then an alternative trial design might be used: HIV-infected individuals could be excluded from randomization to the TB intervention but entered into a parallel PK cohort to evaluate the DDI. Once the potential DDI has been resolved, including by testing different drug dosing, randomization of HIV-infected individuals might proceed expeditiously. Alternatively, an observational study could be conducted whereby HIV-infected people who are on a targeted HIV drug and start a TB drug of interest would undergo PK/PD evaluations. IMPAACT P1026s (NCT00042289) uses this design to evaluate routinely used dosing of ART and TB (DS and DR TB) drugs during pregnancy in HIV-infected and uninfected women. The key is to have an ongoing, approved protocol in place that allows for targeted drugs to be studied without needing to develop a new study for each potential DDI. Irrespective of the design used, the respective advantages and disadvantages of intensive versus sparse drug sampling should be considered to facilitate rapid enrollment and availability of information about potential DDIs.

## Conclusions

TB therapeutic trials that exclude key populations are often not followed by trials in those populations. Pregnant and lactating women, children, and HIV-infected persons contribute a large proportion of the global TB burden and require optimized TB treatment and access to the latest therapeutic advances. Overall, adequate inclusion and appropriate study of these populations remain problematic, particularly for pregnant and lactating women; some advances are being made for children, yet pediatric TB trials lag far behind adult trials despite the potential for better TB treatment outcomes among children, and further evaluation of DDIs is needed in HIV–TB-coinfected populations to ensure that HIV-infected persons, particularly those with more advanced HIV disease, more fully benefit from therapeutic advances. Importantly, despite the differences among these populations, several cross-cutting themes exist and can serve as a way forward toward inclusion of key populations in TB clinical trials (**Box 2**).

**Box 2. Summary of recommendations and cross-cutting issues among key populations**

1. Pregnant and lactating women, children, and HIV-infected persons have increased susceptibility to TB and variable responses during TB treatment, which cannot be predicted by modeling data alone. Inclusion into clinical trials—especially Phase IIb and beyond—is often the best way to generate population-specific data, as postmarketing studies are not prioritized and cause delay in obtaining needed information.
2. Ethics are not a reason to exclude people from clinical trials, but careful consideration of design and involvement of content experts, regulatory agency inputs, and community participation is critical to ensure appropriate trial design and implementation. Inclusion will continue to require careful risks and benefits assessments, weighing direct benefits alongside potential risks of adverse effects from interventions on a case-by-case basis. The uncertainty cost of uniform exclusion results in lack of guidance to inform use of these important TB therapies.
3. Design of trials requires careful attention to how safety, risks, and benefits are defined and measured. Novel approaches may be useful, such as desirability of outcome ranking (DOOR)/response adjusted for duration of antibiotic risk (RADAR), a methodology that integrates overall clinical outcome and patient-level risks and benefits and was specifically developed for clinical trials comparing strategies to optimize antibiotic use [76].
4. Rigorous qualitative research is useful to inform trial design and elicit patient, caregiver, and family preferences regarding trial participation and regimens.

## Abbreviations

1HP: 1 month of daily isoniazid and rifapentine
3HP: 3 months of weekly isoniazid and rifapentine
ACTG: AIDS Clinical Trials Group
AECID: Agencia Española de Cooperación Internacional para el Desarrollo (Spanish Agency for International Development Corporation
AFB: acid-fast bacilli
AFD: French Development Agency
ART: antiretroviral therapy
ATT: antituberculosis therapy
CDC: Centers for Disease Control and Prevention
CROI: Conference on Retroviruses and Opportunistic Infections
DDI: drug–drug interaction
DFID: British Department for International Development
DOOR: desirability of outcome ranking
DR: drug-resistant
DS: drug sensitive
EDCTP: European & Developing Countries Clinical Trials Partnership
FDA: Food and Drug Administration
FQ: fluoroquinolone
IMPAACT: International Maternal Pediatric Adolescent AIDS Clinical Trials Network
ICMR: Indian Council of Medical Research
IGRA: interferon gamma release assay
IPT: isoniazid preventive therapy
IRIS: immune reconstitution inflammatory syndrome
IUATLD: International Union Against Tuberculosis and Lung Disease (The Union)
LPV: lopinavir
LTBI: latent TB infection
MDR: multidrug-resistant
MRC: Medical Research Council
MSF: Médecins Sans Frontières
NACO: National AIDS Control Organization
NIAID: National Institute of Allergy and Infectious Diseases
NICHD: Eunice Kennedy Shriver National Institute of Child Health and Human Development
NIH: National Institutes of Health
NIHR: National Institute for Health Research
NRTI: nucleoside reverse transcriptase inhibitor
NNRTI: nonnucleoside reverse transcriptase inhibitor
NVP: nevirapine
OBR: optimized background regimen
PD: pharmacodynamics
PK: pharmacokinetics
PREGLAC: US Federal Task Force on Research Specific to Pregnant Women and Lactating Women
RADAR: response adjusted for duration of antibiotic risk
RR: rifampicin resistant
SDC: Swiss Agency for Development and Cooperation
TB: tuberculosis
TBD: to be determined
TBTC: Tuberculosis Trials Consortium
THINK: TB&HIV Investigative Network
TST+: tuberculin skin test positive
USAID: United States Agency for International Development
WHO: World Health Organization
XDR: extremely drug-resistant
